# Antihypertensive Effects of Sacubitril/Valsartan Versus Olmesartan: An Updated Systemic Review and Meta-Analysis of Randomized Controlled Trials

**DOI:** 10.7759/cureus.48692

**Published:** 2023-11-12

**Authors:** Roomi Raja, Sandhya Kumari, Muhammad Umer Khan, Araib Ayaz, Duaa Jaffar, Zain Mohamad, Muhammad Ali Muzammil, Nasira Sohail, Saad Ahmed Qureshi, Hamid Saeed, Muhammad Fahad Amin, Ansar Jawad, Giustino Varrassi, Satesh Kumar, Mahima Khatri, Areeba Maryam

**Affiliations:** 1 Medicine, Ziauddin University, Karachi, PAK; 2 Medical College, Ziauddin University, Karachi, PAK; 3 Medicine, Shaheed Mohtarma Benazir Bhutto Medical College Lyari, Karachi, PAK; 4 Medicine, Dearborn High School, Dearborn, USA; 5 Internal Medicine, Dow University of Health Sciences, Karachi, PAK; 6 Medicine, Avicenna Medical and Dental College, Lahore, PAK; 7 Pain Medicine, Paolo Procacci Foundation, Rome, ITA; 8 Medicine and Surgery, Shaheed Mohtarma Benazir Bhutto Medical College, Karachi, PAK; 9 Medicine and Surgery, Dow University of Health Sciences, Karachi, PAK; 10 Emergency Medicine, Holy Family Hospital, Rawalpindi, PAK

**Keywords:** meta-analysis, elderly population, sacubitril/valsartan, hypotension, anti-hypertensives

## Abstract

Sacubitril/valsartan is a drug commonly prescribed for the management of hypertension. However, the complete understanding of its efficacy and safety as an antihypertensive agent remains a subject of ongoing investigation. To address this gap, a meta-analysis was conducted to assess and compare the efficacy and safety of sacubitril/valsartan in relation to olmesartan, an angiotensin receptor blocker (ARB). A thorough search of PubMed, Google Scholar, and Cochrane databases was performed to identify relevant randomized controlled trials (RCTs) and observational studies that could contribute to this meta-analysis. The selected studies were evaluated for their efficacy and safety parameters, including mean sitting and ambulatory blood pressure measurements, common side effects, adverse events, and drug discontinuation rates. A total of eight studies, involving 4488 hypertensive patients, were included in this analysis. Among the participants, 63.5% were administered sacubitril/valsartan, while 36.5% received olmesartan. The analysis revealed significant changes in mean sitting systolic blood pressure (MsSBP), mean sitting diastolic blood pressure (MsDBP), and mean sitting pulse pressure (MsPP) favoring sacubitril/valsartan, with p-values <0.00001, 0.07, and <0.00001, respectively. Additionally, sacubitril/valsartan demonstrated a significant reduction in mean ambulatory systolic blood pressure (MaSBP), mean ambulatory diastolic blood pressure (MaDBP), and mean ambulatory pulse pressure (MaPP) with p-values of 0.001, 0.001, and 0.02, respectively. However, it is important to note that safety outcomes indicated that sacubitril/valsartan was associated with slightly less favorable results compared to olmesartan. This meta-analysis highlights that sacubitril/valsartan exhibits superior efficacy in reducing blood pressure parameters compared to olmesartan in hypertensive patients. Nevertheless, its safety profile appears to be slightly less favorable. To reinforce these findings and provide more robust evidence, further studies with larger sample sizes should be conducted in the future. This comprehensive review serves as a valuable resource for healthcare professionals and researchers seeking to make informed decisions regarding antihypertensive treatment options.

## Introduction and background

Hypertension, commonly acknowledged as increased blood pressure, is a persistent medical ailment distinguished by systolic blood pressure measurements of ≥ 140 mmHg and diastolic blood pressure measurements of ≥ 90 mmHg in two distinct instances. This syndrome substantially increases the likelihood of developing heart and kidney damage, ultimately resulting in serious health consequences, such as heart failure, myocardial infarctions, cerebrovascular accidents, and renal failure, frequently terminating premature fatality [1]. Hypertension presents a noteworthy issue in global health, impacting a considerable proportion of the populace. In 2010, about 1.39 billion individuals, accounting for 31.1% of adults worldwide, encountered the adverse consequences of this condition. The occurrence of this phenomenon is significantly more pronounced in nations with lower and middle levels of income, where around 31.5% of the population, equivalent to almost 1.04 billion individuals, are affected, in contrast to 28.5% (349 million) in countries with higher levels of wealth [2]. Hypertension, characterized by its covert manifestation and often an absence of symptoms, has been commonly referred to as a "silent killer" [1].

In light of the increasing prevalence of cardiovascular and cerebrovascular diseases linked to hypertension, a range of interventions have been implemented to control blood pressure effectively. These interventions include lifestyle modifications, sodium restriction, and medications to reduce blood pressure [3]. Nevertheless, it is essential to acknowledge that a significant proportion of individuals with hypertension do not obtain satisfactory control of their blood pressure when treated with a single antihypertensive medication. As a result, it becomes necessary to prescribe two antihypertensive drugs from different classes to manage the condition [4] effectively. The American College of Cardiology (ACC) and the American Heart Association (AHA) have issued recommendations regarding the initial pharmacological treatment options for hypertension. These guidelines advocate the use of four distinct drug classes as first-line therapies: thiazide diuretics, calcium antagonists, angiotensin-converting enzyme (ACE) inhibitors, and angiotensin receptor blockers (ARBs) [5].

Among the several options, olmesartan, frequently prescribed as an ARB II, exhibits some shared attributes with other members of its pharmacological class and is widely regarded as the most potent. Nevertheless, recent studies have brought to light the significance of sacubitril/valsartan (LCZ696), an innovative angiotensin receptor neprilysin inhibitor (ARNI) that effectively inhibits both neprilysin and angiotensin-2 receptor-1. Multiple studies have indicated that sacubitril/valsartan may provide enhanced effectiveness in reducing high blood pressure when compared to ARBs [6-8]. However, there still needs to be more in our understanding of the safety and long-term effectiveness of sacubitril/valsartan as a medication for high blood pressure. This is especially important considering the concerns about the prolonged inhibition of cerebral neprilysin and its potential effects on the deposition of beta-amyloid and neural toxicity in patients who are receiving long-term antihypertensive treatments. Recent research suggests that the neurotoxic effects of sacubitril/valsartan are not clinically significant when administered at authorized therapeutic levels ranging from 100 to 400 mg per day [6]. As a result, the Food and Drug Administration (FDA) has approved using this treatment in individuals diagnosed with chronic heart failure who display reduced ejection fraction (HFrEF) at New York Heart Association (NYHA) class 2 and higher. The decision was made in light of the notable efficacy of the treatment in reducing both hospitalizations and mortality rates, as evidenced by the findings of the PARADIGM-HF trial [8].

Given the limited research exploring the possibility of sacubitril/valsartan as a treatment for hypertension, our analysis revealed a need for randomized controlled trials (RCTs) and observational studies with inconsistent results. To bridge this gap in information, we conducted a thorough meta-analysis and systematic review to thoroughly evaluate the efficacy and safety of sacubitril/valsartan as a treatment for hypertension.

## Review

Materials and methods

This meta-analysis was carried out in accordance with the Preferred Reporting Items for Systematic Reviews and Meta-Analyses (PRISMA) [9].

Search Strategy

A thorough review of the existing literature was undertaken to identify pertinent papers from the commencement of this research project until July 30, 2022. Systematic queries were conducted on databases such as PubMed (Medline) and Cochrane. To incorporate grey literature and preprints into the study, supplementary searches were conducted on ClinicalTrials.gov, Google Scholar, and medRxiv. A comprehensive search strategy was developed with careful consideration of Medical Subject Headings (MeSH) terms and relevant keywords. The strategy involved the integration of phrases such as "anti-hypertensive," "sacubitril/valsartan," "angiotensin receptor blocker (ARB)," and "elderly" or "aged." It is worth noting that the search results were not subjected to any form of filtering or limits. To enhance comprehension, Google's translation functionality was utilized for non-English language publications. To guarantee the incorporation of relevant research, a comprehensive screening procedure was implemented. Two reviewers, RR and MUK, conducted a thorough evaluation of the titles, complete texts, and abstracts of the publications that were obtained. Afterward, pertinent research was imported into Endnote X9, a software developed by Clarivate Analytics in the United States, to streamline the process of systematically removing duplicate entries.

Eligibility Criteria

Inclusion criteria: The selection of studies for inclusion in this analysis was based on specific criteria, encompassing language, study design, patient demographics, intervention parameters, comparative factors, outcomes of primary interest, and precise definitions. Our inclusion criteria were limited to studies published in the English language. We exclusively considered completed randomized clinical trials as the chosen study design to conduct subsequent meta-analyses. The target patient population under scrutiny consisted of elderly individuals with a confirmed diagnosis of hypertension. The exposure variable of interest was the use of sacubitril/valsartan in hypertensive patients aged over 55 years. In the comparative context, we compared this group to hypertensive patients in the same age bracket who were using other reference drugs, namely, angiotensin-converting enzyme inhibitors (ACEi), angiotensin receptor blockers (ARB), calcium channel blockers (CCB), or beta blockers. Our primary focus revolved around several outcomes, including the impact on the central nervous system, alterations in electrolyte levels, uric acid concentrations, gastrointestinal symptoms, occurrences of upper respiratory tract infections, reports of back pain, changes in systolic and diastolic blood pressure, monitoring of adverse events, as well as assessments of alanine aminotransferase and blood bilirubin levels, and the documentation of drug-related adverse events.

Exclusion criteria: To uphold the rigor and integrity of this meta-analysis, stringent exclusion criteria were meticulously established. Excluded from consideration were studies not documented in English, duplicated research, non-randomized investigations, review articles, and editorials, as these were not deemed suitable for our analytical purposes. Additionally, animal studies and individual case reports were deliberately omitted from our analysis due to their distinct nature and limited applicability to our research objectives. Furthermore, we vigilantly excluded duplicate publications, ensuring that only unique and pertinent research findings were included in the analysis. These meticulous exclusion criteria were employed to safeguard this meta-analysis's methodological soundness and overall quality.

**Figure 1:**
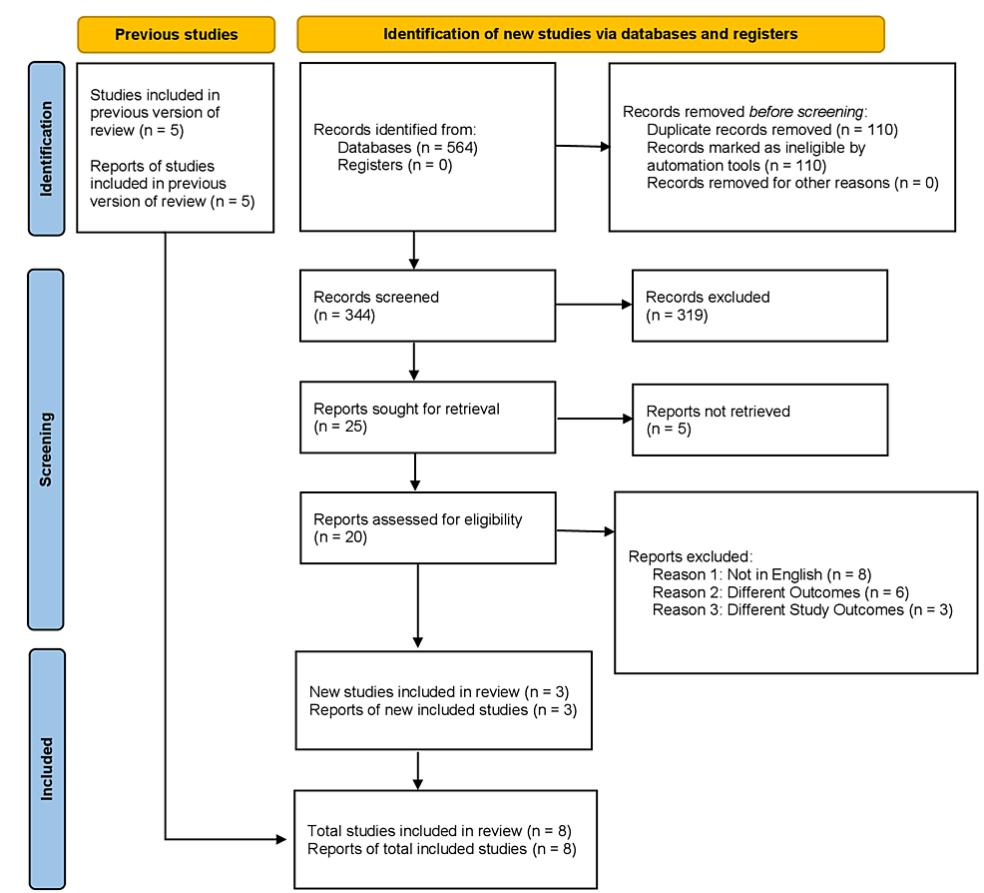
PRISMA flow chart for the included studies PRISMA: Preferred Reporting Items for Systematic Reviews and Meta-Analyses.

In addition, all included RCTs were evaluated based on the 25-item Consolidated Standards of Reporting Trials (CONSORT) checklist, which emphasizes describing how the trials were conceived, analyzed, and interpreted. The quality of the included RCTs was evaluated based on the number of the 25 items that were reported. A correlation exists between the number of reported items and the quality of an RCT. High-quality research will report all 25 criteria. It is illustrated in Figure 1 below.

Data Extraction

To ensure the thorough inclusion of studies, a collaborative effort was undertaken by two researchers, RR and MUK. The selected studies were assessed independently by the researchers, who determined their inclusion or exclusion. In situations characterized by ambiguity or disagreement, a thorough and inclusive discussion was initiated, resulting in the resolution of any contentious matters. A standardized data extraction procedure was utilized to collect relevant information from each included study. The relevant variables extracted included important information such as the name of the first author, the year of publication, the country where the study was conducted, the average age of the participants, the duration of the study, the duration of hypertension, the prescribed treatment for managing hypertension, the use of statins, the drugs used for comparison, and the specific doses of sacubitril/valsartan used. The systematic approach employed for data extraction greatly facilitated a thorough comprehension of the key characteristics of each study.

Study Quality Assessment

The modified Cochrane Collaboration's risk of bias tool was used to assess the quality of published RCTs [10].

Statistical Analysis

In this investigation, we have employed Review Manager 5.4, a software developed by the Cochrane Collaboration (London, UK), to facilitate our meta-analysis. Continuous outcomes have been meticulously presented with mean values and standard deviations. For dichotomous outcomes, we have meticulously extracted relative risks (RRs) and their associated 95% confidence intervals (CIs). The computation of weighted mean differences (WMDs) and the amalgamation of relative risks have been performed by employing the generic inverse-variance and continuous outcome functions within the framework of a random-effects model. Significance was discerned by assessing the p-value, with statistical significance defined as a p-value less than or equal to 0.05. To evaluate publication bias, we conducted a comprehensive assessment employing funnel plots for all the outcomes. Heterogeneity among the diverse trials was quantified and presented as a percentage using the I2 statistic. A value of 25% indicated low heterogeneity, while moderate heterogeneity was defined in the range of 25% to 50%. High heterogeneity was ascribed to instances where the I2 value exceeded 50%. In cases where studies displayed a notable degree of heterogeneity, we implemented sensitivity analyses to discern the impact of each study on the pooled estimate.

Results

Study Selection

A total of 259 articles were identified from the preliminary literature search. After eliminating duplicated articles and based on title and abstract, a total of eight studies were included in this meta-analysis [11-18].

Baseline Characteristics

This study encompassed 4488 participants, all meeting the clinical criteria for hypertension. Within this cohort, 2851 individuals were administered sacubitril/valsartan, while 1637 received olmesartan as part of their antihypertensive therapy. It is noteworthy that the primary antihypertensive agents utilized in these investigations exhibited variability, with variations observed in dosages and administration timings across the studies. Among the eight RCTs, six adhered to the rigorous double-blind, placebo-controlled design [12-14,16-18], while the remaining two studies did not incorporate a control group in their experimental design [11,15]. Furthermore, it is essential to highlight that out of the eight studies, five incorporated a washout period as an integral component of their study protocol [11-14,17]. To facilitate a comprehensive understanding of the research, Tables 1, 2 have been meticulously curated to provide a detailed exposition of the baseline characteristics of the participants, their prevalent medical conditions, pertinent laboratory parameters, and the array of medications employed within the scope of this investigation.

**Table 1:** Baseline characteristics of included studies N: number; SD: standard deviation; RCT: randomized controlled trial; BMI: body mass index; N/A: not available.

Study	Total patients	Study design	Number of patients		Age (mean + SD)		BMI (kg/m2) (mean + SD)		Male, N (%)	
			Sacubitril/valsartan	Olmesartan	Sacubitril/valsartan	Olmesartan	Sacubitril/valsartan	Olmesartan	Sacubitril/valsartan	Olmesartan
Wang et al. (2016) [11]	72	RCT	36	36	55.7 (12.5)	58.9 (7.5)	26.4 (3.8)	25.7 (2.9)	23 (64)	23 (64)
Supasyndh et al. (2017) [12]	588	RCT	296	292	70.5 ± 4.67	70.9 ± 4.67	24.3 ± 3.15	24.6 ± 3.24	142 (48.0)	152 (52.1)
Williams et al. (2017) [13]	454	RCT	229	225	68.2 (5.73)	67.2 (5.97)	28.6 (4.47)	29.1 (4.9)	119 (52.0)	118 (52.4)
Schmieder et al. (2017) [14]	114	RCT	57	57	60.5 ± 7.8	59.2 ± 13.1	28.1 ± 4.5	28.6 ± 3.9	37 (64.9)	40 (70.2)
Izzo Jr et al. (2017) [15]	285	RCT	285	N/A	61	N/A	27.9	N/A	N/A	N/A
Huo et al. (2018) [16]	963	RCT	479	484	57.5 ± 10.17	57.4 ± 10.14	26.4 ± 3.91	26.4 ± 3.92	252 (52.6)	261 (53.9)
Cheung et al. (2018) [17]	375	RCT	188	187	57.1 ± 10.19	58.0 ± 9.09	30.5 ± 5.86	30.6 ± 5.09	N/A	N/A
Rakugi et al. (2022) [18]	1161	RCT	387	389	57.9 ± 10.9	59.6 ± 10.5	25.4 ± 3.7	25.6 ± 3.8	264 (68.2)	286 (73.5)

**Table 2:** Baseline blood pressure values SD: standard deviation; SBP: systolic blood pressure; DBP: diastolic blood pressure; PP: pulse pressure; LCZ696: sacubitril-valsartan; OLM: olmesartan; MsPP: mean sitting pulse pressure; MaPP: mean ambulatory pulse pressure.

Study	Mean sitting SBP, mm Hg (mean ± SD)		Mean sitting DBP, mm Hg (mean ± SD)		MsPP, mm Hg (mean ± SD)		Mean ambulatory SBP, mm Hg (mean ± SD)		Mean ambulatory DBP, mm Hg (mean ± SD)		MaPP, mm Hg (mean ± SD)	
	LCZ696	OLM	LCZ696	OLM	LCZ696	OLM	LCZ696	OLM	LCZ696	OLM	LCZ696	OLM
Supasyndh et al. (2017) [12]	-22.71 ± 14.84	-16.11 ± 14.84	12.9 ± 1.05	-6.49 ± 8.015	-14.21 ± 15.143	-9.76 ± 15.143	-14.23 ± 9.424	-9.14 ± 9.424	-6.95 ± 5.219	-4.47 ± 5.219	-8.19 ± 5.12	-4.62 ± 5.12
Williams et al. (2017) [13]	-15.7 ± 15.81	-13 ± 15.81	-7.3 ± 0.612	-6.5 ± 0.612	-8.25 ± 11.58	-6.45 ± 11.58	-13.75 ± 7.943	-11.7 ± 7.943	-8.1 ± 4.628	-6.95 ± 4.628	-5.55 ± 4.63	-4.8 ± 4.63
Schmieder et al. (2017) [14]	0.2 ± 1.485	-1 ± 1.485	12.9 ± 1.05	11.95 ± 1.05	-6.62 ± 0.91	-4.465 ± 0.91	N/A	N/A	N/A	N/A	N/A	N/A
Huo et al. (2018) [16]	-21.075 ± 18.55	-18.5 ± 18.55	-8.45 ± 11.327	-6.86 ± 11.327	-12.64 ± 12.86	-11.25 ± 12.86	-12.415 ± 9.316	-10.26 ± 9.316	-6.58 ± 5.923	-5.61 ± 5.923	-5.88 ± 4.375	-4.58 ± 4.375
Rakugi et al. (2022) [18]	-19.2 ± 12.65	-14.1 ± 14.6	-9 ± 8.35	-6.7 ± 8.4	-10.25 ± 8.5	-7.4 ± 9.9	N/A	N/A	N/A	N/A	N/A	N/A

Quality Assessment and Publication Bias

**Figure 2:**
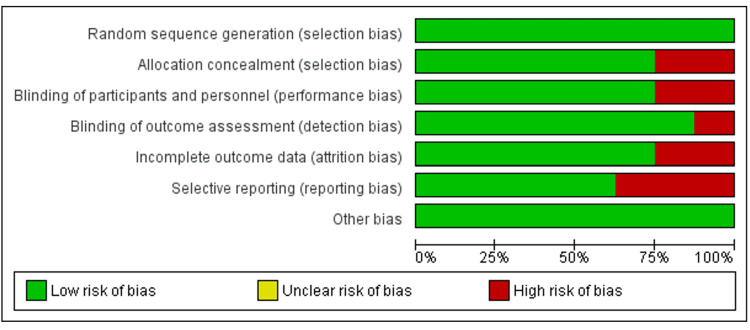
Cochrane risk of bias tool for assessing publication bias in randomized controlled trials

The assessment of RCTs in this study adhered to the rigorous Cochrane methodology, as illustrated in Figure 2. The evaluation was explicitly directed toward trials exhibiting qualities ranging from fair to good. To gauge the risk of bias inherent in the RCTs, the Cochrane risk-of-bias tool version 2 (RoB2) was meticulously applied. This comprehensive tool encompasses several critical domains, namely, random sequence generation, allocation concealment, blinding of participants and outcome assessors, management of incomplete outcome data, and the prevention of selective reporting. It is noteworthy that the majority of the studies subject to this evaluation demonstrated a commendable level of quality as ascertained through their respective risk-of-bias assessments, with most falling within the fair to good quality spectrum. This assessment reflects the rigorous and meticulous nature of the research, as well as the dedication to maintaining high standards in the selection and conduct of the included RCTs.

Outcomes

The Effect on Blood Pressure

**Figure 3:**
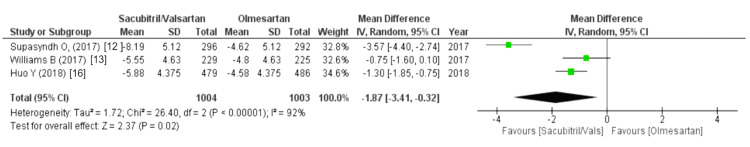
Forest plot of mean ambulatory pulse pressure (MaPP) References [12,13,16].

The effects on blood pressure after administering sacubitril/valsartan vs. olmesartan were assessed using variables like mean sitting systolic blood pressure (MsSBP), mean sitting diastolic blood pressure (MsDBP), mean sitting pulse pressure (MsPP), mean ambulatory systolic blood pressure (MaSBP), mean ambulatory diastolic blood pressure (MaDBP), and mean ambulatory pulse pressure (MaPP). The data on MsSBP (WMD = -3.09 (-6.67, 0.48), p = 0.09, I2 = 95%), MsDBP (WMD = -1.04 (-2.16, 0.07), p = 0.07, I2 = 95%), and MsPP (WMD = -2.29 (-2.91, -1.67), p = <0.00001, I2 = 26%) were outlined by six out of eight studies and demonstrated that treatment with sacubitril/valsartan was associated with a significant reduction in outcomes listed above. For MsSBP and MsDBP, we performed sensitivity analysis by excluding every individual study and thus after removing the study by Schmieder et al. in both outcomes, the change in heterogeneity came to 59% and 73%, respectively [14]. On the other hand, MaSBP (WMD = -3.06 (-4.92, -1.21), p = 0.001, I2 = 81%), MaDBP (WMD = -1.52 (-2.45, -0.60), p = 0.001, I2 = 74%), and MaPP (WMD = -1.87 (-3.41, -0.32), p = 0.02, I2 = 92%) results were reported by three out of eight studies and showed that the sacubitril/valsartan group was associated with a significant reduction in above mentioned outcomes. Similarly, for MaSBP, MaDBP, and MaPP, we performed sensitivity analysis by excluding every study individually, and thus after removing the study by Supasyndh et al., the change in heterogeneity came to 0%, 0%, and 11%, respectively [12]. It is illustrated in Figure 3 below.

The Effects on the Central Nervous System

**Figure 4:**
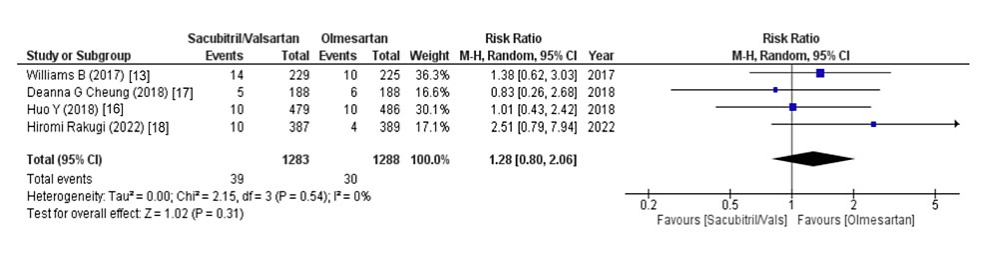
Headache References [13,16-18].

**Figure 5:**
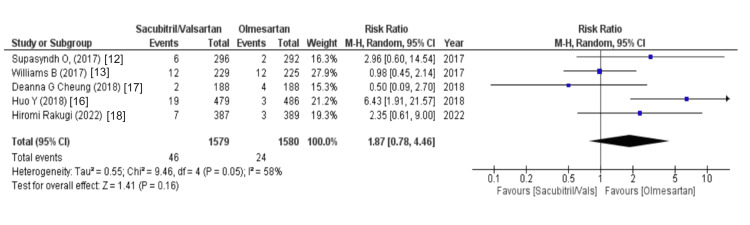
Forest plot of dizziness References [12,13,16-18].

The outcome of anti-hypertensive sacubitril/valsartan on the central nervous system was assessed using headache and dizziness as variables. The data on headache were outlined by four out of eight studies and disclosed that the sacubitril/valsartan group is associated with an increased risk of headache (WMD = 1.28 (0.80, 2.06), p = 0.31, I2 = 0%). Similarly, out of eight studies, five studies reported data on dizziness and revealed that like headache, the sacubitril/valsartan group is also associated with an increased risk of dizziness (WMD = 1.87 (0.78, 4.46), p = 0.16, I2 = 58%), as illustrated in Figures 4, 5.

Effects on Upper Respiratory Tract

**Figure 6:**
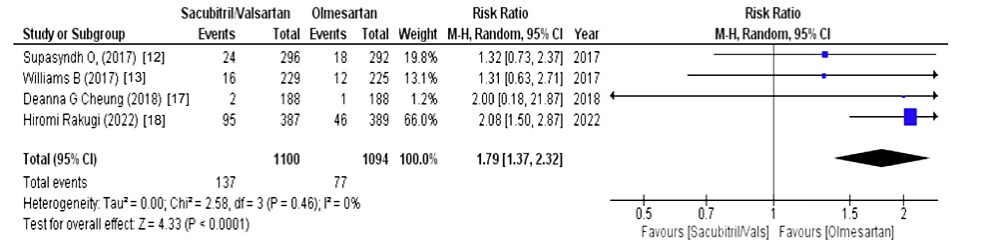
Forest plot of nasopharyngitis References [12,13,17,18].

**Figure 7:**
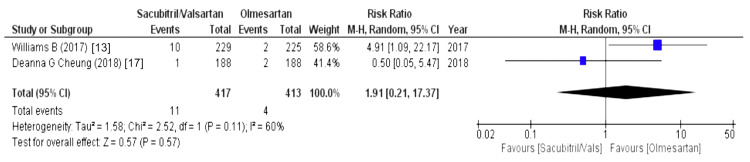
Forest plot of cough References [13,17].

**Figure 8:**
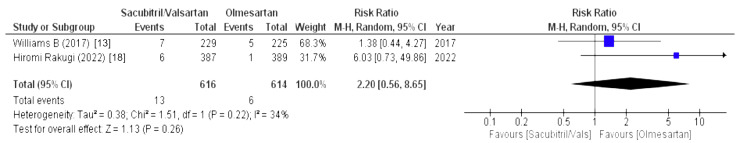
Forest plot of influenza References [13,18].

**Figure 9:**
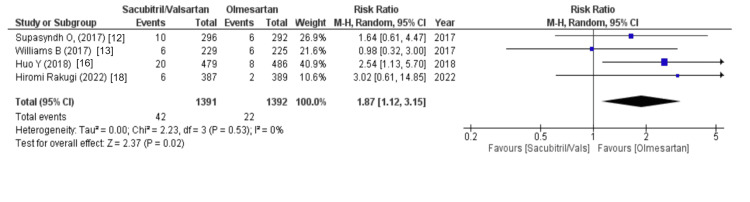
Forest plot of upper respiratory tract infection References [12,13,16,18].

The effect on the upper respiratory tract was assessed using the following variables: nasopharyngitis, cough, influenza, and in some studies upper respiratory tract infection. Above them, nasopharyngitis was outlined by four out of eight studies and revealed that patients receiving sacubitril/valsartan were associated with an increased risk of nasopharyngitis (WMD = 1.79 (1.37, 2.32), p = 0.0001, I2 = 0%). Another variable was cough, which was outlined by two out of eight studies and stated that the olmesartan group was associated with a decreased risk of cough (WMD = 1.91 (0.21,17.37), p = 0.57, I2 = 60%). Similarly, influenza was reported by two out of eight studies and revealed that the sacubitril/valsartan group had a higher risk of influenza (WMD = 2.20 (0.56, 8.65), p = 0.26, I2 = 34%). Lastly, upper respiratory tract infections, as stated by four out of eight studies, were associated with a decreased risk in the olmesartan group (WMD = 1.87 (1.12, 3.15), p = 0.02, I2 = 0%), as illustrated in Figures 6-9.

Effects on Drug and Serious Adverse Events

**Figure 10:**
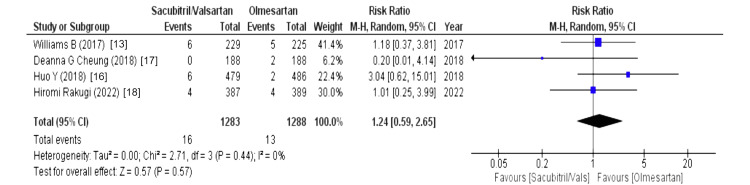
Forest plot of drug-related adverse events References [12,16,17,18].

**Figure 11:**
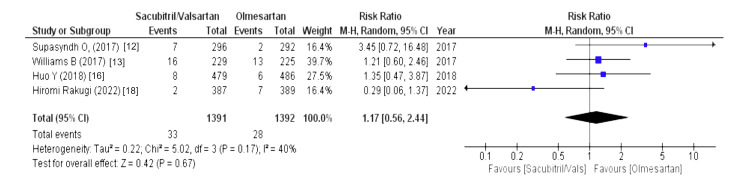
Forest plot of serious adverse events References [12,13,16,18].

**Figure 12:**
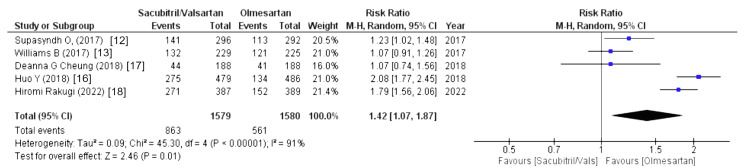
Forest plot of any adverse events References [12,13,16-18].

**Figure 13:**
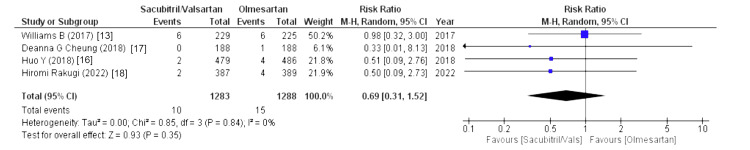
Forest plot of serious adverse events discontinuation References [13,16-18].

**Figure 14:**
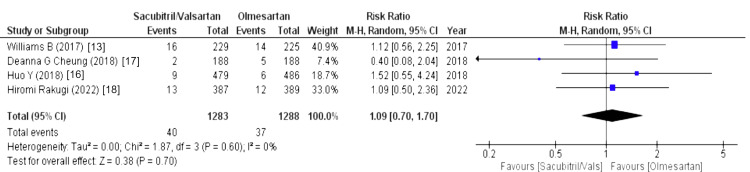
Forest plot of adverse events discontinuation References [13,16-18].

The drug-related and serious adverse events were assessed using the following parameters: drug-related adverse events, serious adverse events, any adverse events, serious adverse events discontinuation, and adverse events discontinuation. Four out of eight studies reported drug-related adverse events (WMD = 1.24 (0.59, 2.65), p = 0.57, I2 = 0%) and adverse events discontinuation (WMD = 1.09 (0.70, 1.70), p = 0.70, I2 = 0%) and pooled analysis demonstrated that the sacubitril/valsartan group is markedly associated with increased risk in above-mentioned outcomes compared to olmesartan. Similarly, five out of eight studies reported that any adverse events were associated with a decreased risk in the olmesartan group (WMD = 1.42 (1.07, 1.87), p = 0.01, I2 = 91%). For any adverse events, we performed sensitivity analysis but there was no difference in heterogeneity after every study was individually excluded, as no single study affected that particular outcome. Lastly, four out of eight studies stated that serious adverse events discontinuation outcome was less pronounced in the sacubitril/valsartan group compared to olmesartan (WMD = 0.69 (0.31, 1.52), p = 0.35, I2 = 0%). Lastly, serious adverse events outcome was reported by four out of eight studies and highlighted that patients in sacubitril/valsartan were highly associated with increased risk in serious adverse events as a comparison to olmesartan (WMD = 1.17 (0.56, 2.44), p = 0.67, I2 = 40%), as illustrated in Figures 10-14.

Effects on Electrolytes

**Figure 15:**
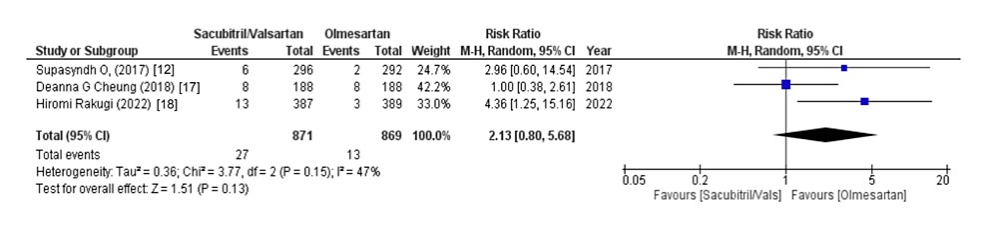
Forest plot of hyperkalemia References [12,17,18].

**Figure 16:**
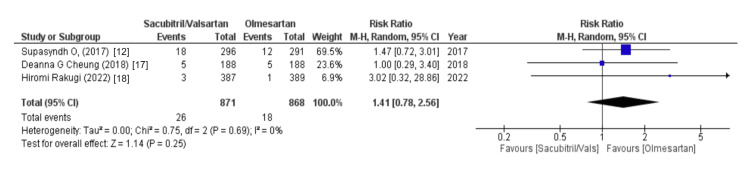
Forest plot of hypokalemia References [12,17,18].

**Figure 17:**
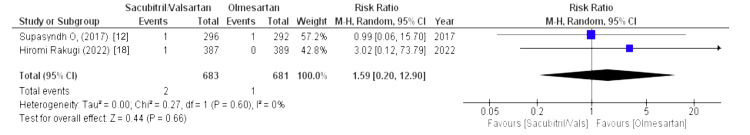
Forest plot of sodium levels References [12,18].

Data containing the values of electrolytes were assessed using variables like hyperkalemia, hypokalemia, and sodium levels. Out of eight, three studies reported data on hyperkalemia and hypokalemia and our forest plots showed that changes in levels of potassium, either hyperkalemia or hypokalemia, were mostly associated with increased risk in sacubitril/valsartan as compared to olmesartan (WMD = 2.13 (0.80, 5.68), p = 0.13, I2 = 47% and WMD = 1.41 (0.78, 2.56), p = 0.25, I2 = 0%, respectively). Similarly, two out of eight studies shared data on sodium levels and our forest plots analyzed that the sacubitril/valsartan group was most likely to have an increased risk of sodium levels less than 130 mmol/l (WMD = 1.59 (0.20, 12.90), p = 0.66, I2 = 0%), as illustrated in Figures 15-17.

Effects on the Gastrointestinal System

**Figure 18:**
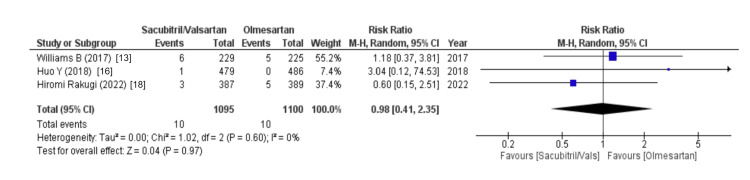
Forest plot of effects on the gastrointestinal system References [13,16,18].

The effects on the gastrointestinal system were assessed using variables like diarrhea and abdominal pain. Diarrhea was proposed by three out of eight studies and the risk of occurrence of diarrhea between sacubitril/valsartan and olmesartan group did not show any statistical significance (WMD = 0.98 (0.41, 2.35), p = 0.97, I2 = 0%). Similarly, two out of eight studies reported abdominal pain as an outcome and revealed that the sacubitril/valsartan group was highly associated with an increased risk of abdominal pain (WMD = 1.19 (0.05, 27.07), p = 0.91, I2 = 65%), as illustrated in Figure 18.

Effect on Low Blood Pressure

**Figure 19:**
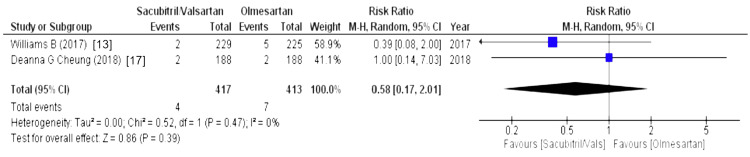
Forest plot of effects on low blood pressure References [13,17].

The effect on blood pressure was evaluated using parameter hypotension. Hypotension was highlighted by two out of eight studies and our forest plot concluded that the sacubitril/valsartan group was more associated with a risk of experiencing hypotension as compared to olmesartan (WMD = 0.58 (0.17, 2.01), p = 0.39, I2 = 0%), as illustrated in Figure 19.

Effect on Blood Bilirubin Level

**Figure 20:**
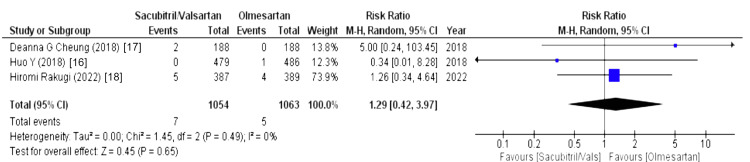
Forest plot of effect on blood bilirubin level References [16-18].

Data on blood bilirubin levels was presented by three out of eight studies and was assessed using levels of blood bilirubin. Their forest plot revealed that there was more association of risk of increased levels of bilirubin in blood in the sacubitril/valsartan group (WMD = 1.29 (0.42, 3.97), p = 0.65, I2 = 0%), as illustrated in Figure 20.

Effect on Back Pain

Only two out of eight studies reported data on back pain and their forest plot analysis proposed that the chances of occurrence of back pain in either group did not show any statistical significance (WMD = 1.06 (0.07, 17.28), p = 0.96, I2 = 80%).

Effect on Uric Acid Level

**Figure 21:**
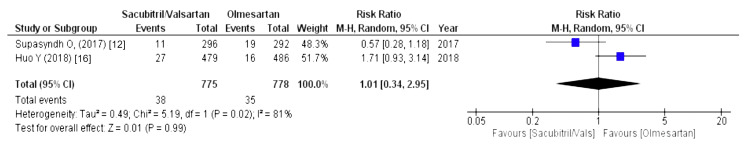
Forest plot of effect on the uric acid level References [12,16].

The data on the uric acid level were presented by only two studies and their analysis revealed no statistical significance between sacubitril/valsartan and olmesartan group (WMD = 1.01 (0.34, 2.95), p = 0.99, I2 = 81%), as illustrated in Figure 21.

Effect on Alanine Aminotransferase

**Figure 22:**
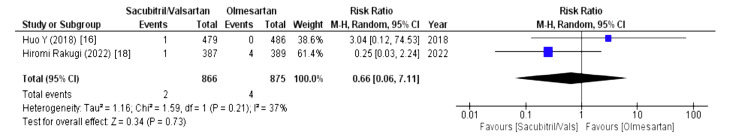
Forest plot of effect on alanine aminotransferase References [16,18].

Alanine aminotransferase levels were reported by two out of eight studies and their forest plot analysis disclosed that treatment with sacubitril/valsartan is associated with an increased risk of decrease in levels of alanine aminotransferase as compared to olmesartan (WMD = 0.66 (0.06, 7.11), p = 0.73, I2 = 37%), as illustrated in Figure 22.

Discussion

In this discussion, we present a comprehensive evaluation of the comparative efficacy and safety of two antihypertensive agents, sacubitril/valsartan (ARNI) and olmesartan (ARB). The study undertaken herein involved a syntactic representation designed to scrutinize the effects of these medications, with the overarching goal of discerning their relative merits. Sacubitril/valsartan was juxtaposed with olmesartan in a rigorous examination of their efficacy and safety profiles. The results unequivocally revealed that sacubitril/valsartan demonstrated superior outcomes in reducing both mean systolic and diastolic blood pressure. These findings underscore the heightened efficacy of sacubitril/valsartan when compared to olmesartan [19].

The unique pharmacological properties of sacubitril, functioning as a neprilysin inhibitor, are paramount to its antihypertensive effects. When administered with the angiotensin receptor blocker, valsartan, a synergistic inhibitory effect is achieved through the simultaneous inhibition of neutral endopeptidase and angiotensin II receptors [20]. This combination confers greater hemodynamic and neurohormonal benefits compared to using olmesartan in isolation [21].

The influence of B-type natriuretic peptide (BNP), a crucial protein hormone in circulatory regulation, should be considered. Sacubitril/valsartan enhances the endogenous levels of BNP, thereby reducing ventricular hemodynamic loading and left ventricular end-diastolic pressure. This cardiocirculatory modulation, amplified by sacubitril's inhibition of BNP degradation, underscores the unique cardioprotective attributes of this combination therapy [22]. The heart's role in maintaining cardiocirculatory hemostasis has garnered newfound significance with the advent of sacubitril/valsartan, which incorporates natriuretic, diuretic, vasodilating, anti-adrenergic, and anti-apoptotic properties [23-25].

Our meta-analysis delved into various parameters to elucidate outcome differences between the two study groups, namely, sacubitril/valsartan and olmesartan. Eight RCTs, encompassing 4488 hypertensive patients, were rigorously reviewed. Among these patients, 2851 were assigned to the sacubitril/valsartan group, while the remaining 1637 received olmesartan. The investigation scrutinized the effects of the medications on adverse events, serious adverse events, and discontinuations due to these events. It became apparent that these outcomes were more frequently associated with sacubitril/valsartan.

Furthermore, the vasodilatory and antihypertensive properties of sacubitril/valsartan and olmesartan were thoroughly explored and compared against the results of previous meta-analyses. Parameters of interest included mean sitting systolic blood pressure, mean sitting diastolic blood pressure, mean sitting pulse pressure, mean ambulatory systolic blood pressure, mean ambulatory diastolic blood pressure, and mean ambulatory pulse pressure. Sacubitril/valsartan consistently exhibited a significantly superior antihypertensive effect in both sitting and ambulatory blood pressure measurements, with a more pronounced impact on diastolic pressure than olmesartan. These findings were consistent with prior research, reaffirming the enhanced efficacy of sacubitril/valsartan, albeit with some additional side effects.

Regarding side effects, the sacubitril/valsartan group exhibited a higher incidence of adverse events related to the central nervous system, upper respiratory tract infections, and gastrointestinal issues. The study also analyzed the effects on electrolytes, particularly potassium and sodium levels. Sacubitril/valsartan was associated with disturbances in potassium levels, manifesting as hyperkalemia or hypokalemia, as well as reduced sodium levels, indicating hyponatremia.

This meta-analysis has several advantages compared to previous studies. We incorporated three additional randomized control trials, substantially increasing the sample size and bolstering the robustness of our findings. Rigorous statistical methods, including various tests and plots for publication bias, sensitivity analysis for heterogeneous studies, and parameters such as side effects, serious adverse events, and electrolyte disturbances, added depth to our investigation.

Nonetheless, it is imperative to acknowledge certain limitations. Clinical heterogeneity stemming from variations in study designs, sample sizes, interventions, and patient characteristics, including ethnicity, age, and body mass index, introduced some degree of heterogeneity in the results. Discrepancies in the trial characteristics further contributed to this heterogeneity. Variability in follow-up periods and the absence of placebo groups in some studies added complexity to the analysis. Dosing information for control groups was often lacking in the included studies. Additionally, some trials incorporated different doses of sacubitril/valsartan and olmesartan, which may have impacted the results. Despite these limitations, the overall robustness of our study findings and the addition of novel parameters justify its contribution to the body of knowledge in this field.

## Conclusions

In summary, our meta-analysis indicates that sacubitril/valsartan exhibits significant promise as an effective treatment for hypertension, demonstrating favorable outcomes in blood pressure reduction with a manageable side effect profile. With data from 4488 patients across eight randomized control trials, we find consistent evidence that supports the use of sacubitril/valsartan, mainly when administered within a specific dosage range. While the results are promising, further research, including more extensive, high-quality trials, will be invaluable in solidifying the place of sacubitril/valsartan in the management of hypertension.
